# Exploring the preference of devices that can measure movement among people who have been hospitalised: A discrete choice experiment

**DOI:** 10.1177/20552076261454592

**Published:** 2026-06-14

**Authors:** Eliza Becker, Richard Norman, Carol Watson, Meg Harrold, Kylie Hill

**Affiliations:** 1 Curtin School of Allied Health, 1649 Curtin University, Perth, WA, Australia; 2 East CareConnect, East Metropolitan Health Service , Perth, WA, Australia; 3 Curtin School of Population Health, 1649 Curtin University, Perth, WA, Australia; 4 Physiotherapy Department, 6508 Royal Perth Hospital, Perth, WA, Australia

**Keywords:** movement, sensor, hospital, technology, consumers

## Abstract

**Importance:**

Monitoring bodily movement in people admitted to hospital is important for assessing clinical status and preventing complications associated with immobility. Movement sensor systems offer a mechanism to support human assessment by enabling continuous monitoring. Adoption will depend on consumer values and preferences.

**Objective:**

To explore consumer preferences for a hospital-based movement sensor system using a discrete choice experiment (DCE).

**Design, Setting and Participants:**

A DCE survey evaluating seven attributes (time and complexity to set up, accuracy, sensor type, attachment, reusability and number) was completed by 1,040 health consumers in Perth, Western Australia.

**Results:**

Respondents prefer devices that are accurate (only inaccurate 1 in 100 cases versus 1 in 5 cases β = 0.711, p < 0.001) and can be set up quickly (2min versus 15min, β = 0.253, p < 0.001) and independently (versus with support of a professional β = 0.186, p < 0.001). Camera presence in a movement sensor system was less concerning to participants than anticipated. Participants had a small preference for systems without a camera (versus with a camera β = 0.153, p < 0.001) and when a camera was included, had no preference for how footage was displayed to a healthcare professional (identifiable versus non-identifiable footage β = 0.025, p = 0.374).

**Conclusion and Relevance:**

Consumers prioritised accuracy and independence and appear indifferent towards the inclusion of a camera in a movement monitoring system. These findings support a hybrid approach integrating wearable sensors with cameras to achieve high precision without compromising acceptance.

## 1. Introduction

Normal, purposeful bodily movement is a hallmark of health across the lifespan. As such, in a hospital setting, clinicians often use sudden changes in bodily movement as an early warning sign of clinical deterioration. For example, the sudden paralysis of any one body part is indicative of a neurological event.^1^ Sudden increases in non-purposeful movement may suggest seizure activity. Even subtle changes in movement such as fidgeting can suggest an untoward medical complication, such as delirium.^2^ Aside from facilitating early recognition of medical complications, tracking how much a person moves in a hospital bed can help clinicians to respond to the risks associated with extended bed rest.^2^ Specifically, extended periods of immobility can lead to muscle deconditioning and increased risk of deep vein thrombosis and lying in one position can compromise skin integrity and lead to pressure injuries.^3–5^ These complications prolong hospital stay and can have long-term effects on physical function.^6^ Unfortunately, outside specialised hospital wards, such as critical care units, movement in people who are hospitalised is monitored episodically and only by direct observation by a healthcare professional.^7^ The capacity to continuously monitor and interpret changes in movement in a hospital setting may facilitate earlier recognition of medical complications and periods of prolonged recumbency allowing for more timely intervention and improved outcomes.

Currently, the options for monitoring the bodily movement of people admitted to hospital present a difficult trade-off between clinical precision and user independence. At one extreme, kinematic systems offer the ‘gold standard’ for accuracy, yet the reliance on intensive calibration and a high number of individual components makes them labor intensive to set up and unsuitable for the fast-paced environment of a busy ward.^8,9^ More practical, easy to set up solutions such as pressure mattresses with load cells and cameras are accurate with but come with substantial limitations. Specifically, load cells can accurately classify movement when a person is resting in bed, however are unable to detect movement when the person is not in contact with the mattress (e.g. sitting out of bed).^10,11^ Similarly, cameras have been shown to provide accurate information, but only when body parts are not obscured by bed linen (sheets and/or blankets). There are also concerns that cameras will record identifying information and compromise privacy of patients and staff.^12^ At the other end of the spectrum, commercially available wearables (e.g., Apple Watch (https://www.apple.com/au/watch/, Cupertino, CA, USA) or Fitbit (https://store.google.com/au/category/trackers?hl=en-GB, San Francisco, CA, USA)) offer high useability, yet they often lack the precision and kinematic detail required for clinical-grade hospital monitoring.^13^ However, the accuracy of wearable systems can be improved by adding more devices. Specifically, earlier work done by our group has shown that five inertial measurement units (a type of wearable device) produced an accurate classification of postures in bed (i.e. lying prone or supine, side lying) 88% of the time.^14^ Nevertheless, a higher number of wearable device units increases set-up time and raises concerns with infection control with between-person use and the reliance on Bluetooth connectivity to download data.^15,16^ Additionally, as these multi-device systems become more complex, they require efficient authentication processes to protect patient privacy.^17^

Although these options exist, there has been almost no uptake in the hospital setting. This highlights an opportunity to improve alignment between engineering capabilities and user (or consumer) preferences. An understanding of the consumer’s voice is needed to increase the likelihood of the acceptance of a movement sensor system and is a prerequisite for developing a system that is both technically capable and practically viable. There is a dearth of information regarding factors that are likely to influence consumer’s acceptance of these systems in a hospital setting. This study provides quantitative data regarding the preferences of people who have been recently hospitalised regarding the characteristics of a movement sensor system in hospital. These data will provide blueprint of considerations for developers who design future movement sensor systems to optimise the likelihood that they will be accepted by consumers in a hospital setting.

## 2. Methods

We conducted a Discrete Choice Experiment (DCE) which is the most common quantitative method for assessment of consumer preferences^18,19^. This approach involved presenting participants with hypothetical scenarios and offering them a choice between two or more alternatives.^20^ This methodology captures how people make trade-offs between attributes, exploring the relative importance of various factors and how these influence choices. The study was conducted in Perth, Western Australia. Data collection was undertaken between January 2024 and April 2024. This study was performed in accordance with the ethical requirements set out in the Australian National Health and Medical Research Council’s National Statement on Ethical Conduct in Human Research and the principles of the Declaration of Helsinki. Approval was granted by Human Research Ethics Committee at Royal Perth Hospital (HREC20245189) with reciprocal approval from Curtin University (HREC2023-0118).

### 2.1. Role of the funding source

This work was financially supported by the Royal Perth Hospital Research Foundation, Springboard Plus Grant (200/2022). The funder played no role in the design, conduct or reporting of this study.

### 2.2. Preparatory work

To develop a survey to be used for the DCE we undertook preparatory work including a literature review and focus group interviews.

A literature review was conducted to understand factors that have been shown to influence acceptability of movement sensor systems in healthcare settings. The search strategy used for the literature review is available in the online supplement. Papers were reviewed, and recurring themes were extracted to develop an interview schedule to guide the focus group discussions.

Focus groups were run with adults who had been hospitalised in the previous 24 months (n = 3), their nominated carer (n = 3), and healthcare professionals who worked in a hospital and had at least five years of clinical experience (n = 16). Recruitment of consumers and carers was achieved through an open advertisement to people known to a university-run consumer network. Recruitment of health professionals was achieved by emailing the Heads of Departments within local tertiary hospitals for medical, nursing, physiotherapy and occupational therapy.

Focus groups were completed in a 45-minute session. All participants provided informed, written consent prior to focus groups commencing. To provide a context for the interview schedule for each focus group, participants were introduced to a fictitious case study of an older adult admitted after abdominal surgery, resting in bed on a hospital ward. Participants were then asked to draw on their own experiences to answer questions. Visual aids such as images and videos (found online) were used to orientate participants to different technologies available to measure movement and participants were encouraged to share their thoughts about the benefits and shortcomings of each technology. All interviews were audio-recorded and transcribed verbatim. These transcripts were analysed using an abductive approach, in which themes addressed in the interview schedule were explored (deductive) whilst remaining open to new ideas raised during interviews (inductive). This preparatory work aimed to identify attributes (the features of a system) and levels or categories (variations in attributes) for the DCE survey.

Where focus group participants expressed a unanimous preference for specific levels or categories within an attribute, the attribute was considered to have ‘informed priors’, and the levels or categories were ordered from most preferred to least preferred. For example, focus groups identified that for the attribute ‘time to set-up’, people were likely to favor systems that took less time to set-up. Therefore, levels within this attribute for time to set-up were ordered from the shortest duration (most preferred) to the longest duration (least preferred). The final survey comprised seven attributes, each with between two and six associated levels or categories. Three of the seven attributes had informed priors. The seven attributes are summarised in Table 1.

**Table 1.** Attributes and levels to be included in the survey, ordered as they were identified.

Attribute	Description	Levels or categories
Time*	Setting up the movement sensor takes this amount of time.	2 minutes
		5 minutes
		10 minutes
		15 minutes
Complexity*	Setting up the movement sensor is this complex and takes this much external support.	The device is simple to set up. It can be set up by a consumer or carer independently.
		The device is difficult to set up. It must be set up by a health professional
Accuracy*	Number of ‘false alarms’ produced by the movement sensor. A false alarm results in your healthcare provider interrupting you unnecessarily	Inaccurate in 1 out of 5 cases (Interrupted once an hour)
		Inaccurate in 1 out of 10 cases (every 6 hours)
		Inaccurate in 1 out of 50 cases (once every 12 hours)
		Inaccurate in 1 out of 100 cases (once every 24 hours)
Sensor type	In addition to a wearable device, the system can include a camera that captures identifiable or non-identifiable footage, only visible to health care provider	Video camera (identifiable footage) and wearable
		Thermal camera (unidentifiable footage) and wearable
		No camera, just wearable
Attachment type	Method of attaching wearable device to skin.	Stuck with adhesive (requires shaving)
		Stuck with a band
Reusability	A single use device must be discarded after use, a reusable device can be reattached.	Single use
		Reusable
Number of attachments	Number of wearable devices attached to the consumer.	6 attachments (RUL, RLL, LUL, LLL, C, H)
		4 attachments (RUL, RLL, LUL, LLL)
		2 attachments (C, H)
		2 attachments (C, RUL OR LUL)

C – chest, H – head, RLL/LLL – right/left lower limb, RUL/LUL – right/left upper limb.

*indicates attributes where informed priors were used.

### 2.3. Survey design

**Figure 1.**
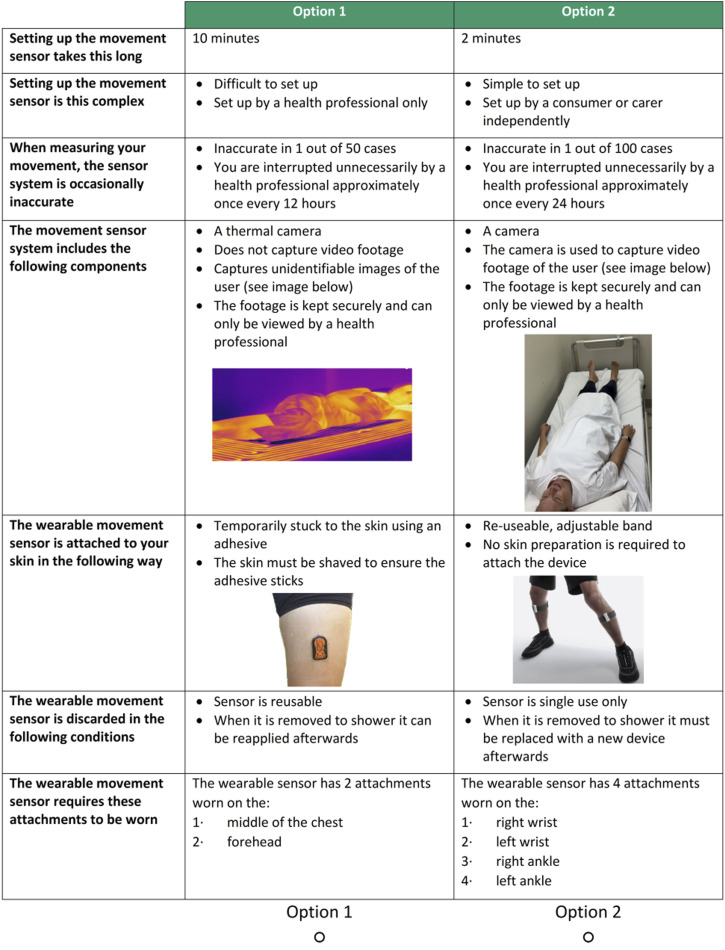
Example choice set

The attributes and levels in our DCE resulted in 1,536 possible response combinations (see online supplement for full details). To manage this, a fractional factorial design was used. This is a common approach in DCEs to ensure that individual surveys comprise a manageable subset of combinations.^20^ This design was generated in Ngene (version 1.4.0, ChoiceMetrics, Sydney, Australia) using a D-efficient algorithm to maximise statistical efficiency and support precise estimation of preferences and resulted in 20 choice sets. A *choice set* is a question in the survey where participants are shown two different scenarios (each made up of different combinations of attribute levels) and asked to choose the one they prefer. An example choice set from this survey is illustrated in Figure 1, and the full survey is available in the online supplement. To further reduce burden, the 20 choice sets were split into two blocks of 10 and participants were randomly assigned to one of two groups. The survey was piloted to ensure it took no more than 15 minutes to complete.

### 2.4. Sample size and sampling strategy

Recruitment was undertaken by a market research company, Qualtrics (https://www.qualtrics.com/en-au/, Utah, USA). This company houses a database of information on people who have previously completed online surveys and incentivises them (e.g. gift cards, airline miles, cash) to complete additional surveys. Qualtrics applies standard data integrity checks such as monitoring routers to minimise duplication, excluding non-sensical or fraudulent responses as well as excluding any data from survey that were completed in less than a third of the median completion time. For this study, participants were selected if they understood written English, were aged 18 years or older and had been admitted to an acute hospital ward in Australia for more than 24 hours in the last five years. At the start of the survey, participants were provided with information on the material (including time to complete and types of questions) and provided consent to participate.

Distribution of the survey was paused after data were collected on 58 participants. Data were carefully interrogated to ensure the there was no missing values and that responses seemed appropriate. Once confirmed, distribution of the survey continued until complete data were available on n = 1,040. This sample size is considerably larger than the previously reported median sample size of published DCEs (n = 394).^19^ Of note, it is also greater that earlier recommendations of 20 to 30 participants per choice set, which allowed us to detect small differences [11].

### 2.5. Data analysis

The choice data were analysed using a multinomial logit model, which is grounded in Random Utility Theory.^19^ This approach assumes that a participant’s choice is driven by a desire to maximise ‘utility’ (or value), allowing us to mathematically separate the influence of specific attributes from random variation in decision-making. This analysis highlights the system attributes and levels that are likely to be meaningful to the whole population. A p-value <0.05 was used to denote statistical significance.

## 3. Results

Regarding the preparatory work, 35 papers were reviewed and the themes addressed in the interview schedule were; types of sensors, privacy and use of any camera, accuracy and the impact on the environment. For this study, we contextualised accuracy as the “number of unnecessary interruptions” or number of false alarms that the device would generate in a 24-hour period. During focus groups healthcare professionals valued “device accuracy,” however consumers expressed concerns regarding the idea of unnecessary interruptions (i.e. false alarms). As the aim of this study was to understand the consumer-voice, we adopted consumer-aligned language to describe accuracy. With knowledge gained from the focus groups, these themes were expanded to form the basis of the DCE survey.

The survey was commenced by 2,170 participants and of these, complete data were available on 1,040 participants. The age of this sample is described in Table 2.

**Table 2.** Age of participants by group.

Age range	Group A	Group B	Total
18 to 24 years	54	55	109 (10%)
25 to 34 years	133	137	270 (26%)
35 to 44 years	125	133	258 (25%)
45 to 54 years	66	75	141 (14%)
55 to 64 years	56	50	106 (10%)
65+ years	79	77	156 (15%)
Total	513	527	1,040 (100%)

The results of the regression analysis are shown in Table 3. All attributes influenced the choice of movement sensor systems. The attribute that had the largest influence was accuracy. Attributes that had a moderate influence were time to set-up and complexity of the set up. Attributes that had the smallest influence were number of attachments, type and reusability.

**Table 3.** Regression analysis.

Attribute	Level	Coefficient	Z score	P value	CI
Time	15 min (reference group)	0	0	​	​
	10 min	0.074	2.000	0.046	0.001 to 0.146
	5 min	0.270	7.110	<0.001	0.196 to 0.345
	2 min	0.253	7.100	<0.001	0.183 to 0.323
Complexity	The device is difficult to set up. It must be set up by a health professional (reference group)	0	0	​	​
	The device is simple to set up. It can be set up by a consumer or carer independently	0.186	8.540	<0.001	0.144 to 0.229
Accuracy	Inaccurate in 1 out of 5 cases (Interrupted once an hour) (reference group)	0	0	​	​
	Inaccurate in 1 out of 10 cases (every 6 hours)	0.203	5.580	<0.001	0.132 to 0.275
	Inaccurate in 1 out of 50 cases (once every 12 hours)	0.505	14.290	<0.001	0.436 to 0.575
	Inaccurate in 1 out of 100 cases (once every 24 hours)	0.711	19.290	<0.001	0.639 to 0.783
Sensor Type	Video camera (identifiable footage) and wearable (reference group)	0	0	​	​
	Thermal camera (unidentifiable footage) and wearable	0.025	0.890	0.374	-0.030 to 0.080
	No camera, just wearable	0.153	4.810	<0.001	0.091 to 0.215
Attachment (type)	Stuck with adhesive (requires shaving) (reference group)	0	0	​	​
	Stuck with a band	0.036	1.710	0.086	-0.005 to 0.077
Reusability	Single use (reference group)	0	0	​	​
	Reusable	0.125	5.900	<0.001	0.083 to 0.166
Attachments (no.)	6 attachments (RUL, RLL, LUL, LLL, C, H) (reference group)	0	0	​	​
	4 attachments (RUL, RLL, LUL, LLL)	0.126	2.840	0.005	0.039 to 0.213
	2 attachments (C, H)	-0.057	-1.340	0.180	-0.141 to 0.026
	2 attachments (C, RUL or LUL)	-0.045	-0.990	0.322	-0.133 to 0.044
	1 attachment (C)	0.155	3.570	<0.001	0.070 to 0.240

C – chest, CI – Confidence Interval, H – head, RLL/LLL – right/left lower limb, RUL/LUL – right/left upper limb.

## 4. Discussion

This study contributes a set of attributes that are likely to influence acceptance of a movement sensor system in adults who were hospitalised and quantified how these people make trade-offs between attributes. The results suggests that there were seven key attributes that are likely to influence acceptance; in order of importance to the participant these were accuracy, time, complexity to set-up, presence of a camera, type of sensor, number of attachments and reusability. Surprisingly, participants were somewhat ambivalent to the inclusion of a camera in a movement monitoring system. Our findings highlight that consumers value an accurate device that they can use independently and are likely to accept a camera as part of a movement monitoring system.

Our finding that device accuracy was valued highly by participants is perhaps not surprising and reinforces the need to design highly precise monitoring systems. The accuracy of a movement monitoring system can be conceptualised in two ways. The first is whether or not the system can correctly identify an important movement (specificity). This has been the focus on earlier work in this area.^14^ The second is whether or not the system can ignore movements that are not considered important (sensitivity) and this was the measurement property focused on in this study. People who are hospitalised as well as healthcare professionals have a low tolerance for low sensitivity monitoring system.^21^ That is, interruption by staff (e.g. routine checks that are completed by nursing staff) and alarm noises have been repeatedly ranked by people who are hospitalised as leading factors that compromise sleep architecture and mood.^21,22^ The other concern is that a high number of false positive alarms produces alarm fatigue which increases the risk that significant alarms will be ignored by healthcare professionals.^15,23^ Our data would suggest that prior to being deployed into a hospital setting, consideration is given refining false alarms for movement monitoring systems. This could be achieved using machine learning models^24^ or centralised monitoring teams.^25^

Results of this study show a preference for people who are hospitalised to be able to set up a movement monitoring system quickly and without assistance from healthcare professionals.^26^ This is consistent with earlier work that has explored the relationship between independence and quality of life in specific populations (i.e. people with Parkinson’s Disease and hip fractures) and demonstrated that increased independence is associated with better quality of life.^27,28^ Interestingly, while our data demonstrated that simple devices were valued, this did not align with a preference for a system that uses the least number of sensors. Compared with a comprehensive approach that used 6 sensors (attachments to all four limbs, head and chest), there was no preference for a system with just two sensors on the chest and head, or chest and one upper limb. A movement monitoring system that used four sensors (all four limbs) or a single sensor (to the chest) were preferred. It appears that a system that needed to attach to the head were considered less desirable. This is likely to be because devices that attach to the head may make activities of daily living, such as sitting up in bed, eating and drinking more challenging, or perhaps because such attachments are not discreet.^29^ Sensors that attach to a limb have become widely accepted in the community with many people using them as part of a health living strategy.^30^ It may be that participants in our study perceived sensors attached to the limbs as more familiar and easier to use then those that attach to the head.

We were somewhat surprised by our finding that participants were ambivalent towards the use of camera and video footage being used in a movement monitoring system. This study required participants to consider both the physical presence of a camera at the point of care and also how the image would be displayed to a health professional. Although participants did indicate a preference for a system that had no camera this was far less salient than other preferences for other attributes. Notably, when a camera was part of the system, participants reported no discernable preference for non-identifiable footage over identifiable footage. Outside the hospital setting, earlier work has reported a high level of acceptance for images and camera to be used in healthcare. Specifically, following hand surgery, 97% of people reported being accepting of a surgeon sending photographs of their hand via mobile phone as a way to improve clinical communication.^31^ Indeed, there is also high acceptance of camera systems being used to facilitate telehealth appointments, sleep studies and even to measure vital signs non-invasively.^32,33^ Nevertheless, we had anticipated that during a period of hospitalisation for an acute condition, participants would report a strong preference for a movement monitoring system to have no identifiable camera footage. This is because during such periods, people are vulnerable and may not be fully clothed, can be dishevelled and have fluctuating levels of consciousness. Earlier work has reported the unease that healthcare professionals feel about people in hospital filming with smartphones and sharing videos online 12. The data in our study questions the assumption that people who are admitted to hospital are reluctant to have cameras involved in their care. It may be that when people feel their survival is compromised, they are prepared to accept any form of healthcare monitoring that assists with improving their prognosis. Alternatively, the belief that there is a reluctance to use camera footage in people who are hospitalised may stem from the healthcare professionals themselves. That is, they are uncomfortable with being watched, and potentially scrutinised, as they deliver care. Further work is need to unpack whether staff discomfort, rather than resistance from people who are hospitalised, is driving any reluctance to use these systems to capture movement. Regardless of the reason underpinning the reluctance to use cameras in a hospital setting, The Australian Medical Association has strict rules about how images and film are captured, stored and documented in the patient record.^34^ Our data suggest the possibility of novel path forward in which bodily movement is monitored by combining camera data, which were previously thought to be unviable due to concerns with consumer acceptance, with data from limb-worn sensors. Future research could explore if this approach may allow developers to reduce the number of physical attachments satisfying the consumers desire for an easy-to-use system while using the camera feed to maintain the clinical-grade accuracy typically lost when reducing sensor count.

### 4.1. Limitations

A strength of this study is the large sample included; we had complete data on more than 1,000 participants. A limitation of this study is that it did not ask participants to consider the viewpoint of healthcare professionals or hospital administrators, both of whom are critical in the acceptance of a movement monitoring system. Future research should focus on capturing these viewpoints and the feasibility of applying a movement monitoring system in a hospital setting.

## 5. Conclusion

Using a DCE, this study explored the voice of adults who had been hospitalised on factors that would influence their acceptance of a movement monitoring system in this setting. Seven attributes influenced device acceptance and of these, accuracy of the system and the time and complexity of setting up the system were the most important. This study identifies a significant gap between current movement sensor system capabilities and user acceptance. By demonstrating that users value both high accuracy and quick and easy set up this study suggests that future sensors should consider moving away from complex designs and toward ‘intelligent’ simple systems. For developers, this means focusing on creating systems that can maintain high accuracy even when a device is applied quickly or imperfectly. Our finding that consumers appear to be less concerned by the presence of a camera in a movement monitoring system than expected creates an opportunity to integrate this technology into future systems. Future research should focus on how to build ‘the ideal monitoring solution’ which maximises both clinical accuracy and high user independence, possibly through a hybrid system that combines cameras with other technology. 

## Supplemental material

Supplemental material - Exploring the preference of devices that can measure movement among people who have been hospitalised: A discrete choice experiment Supplemental material for Exploring the preference of devices that can measure movement among people who have been hospitalised: A discrete choice experiment by Eliza Becker, Richard Norman, Carol Watson, Meg Harrold and Kylie Hill in Digital Health

## Data Availability

This research includes sensitive information such as participant data and is therefore not available.*
