# Supplementary material for: Exploring the preference of devices that can measure movement among people who have been hospitalised: A discrete choice experiment
Source: Digit Health. 2026 Jun 14;12:20552076261454592. doi: 10.1177/20552076261454592 (PMC13269976; doi:10.1177/20552076261454592)
Supplement: Supplemental material - Exploring the preference of devices that can measure movement among people who have been hospitalised: A discrete choice experiment [file sj-pdf-1-dhj-10.1177_20552076261454592.pdf]

Exploring the preference of devices that can measure movement among people who have been hospitalised: a discrete choice experiment

Literature review

A literature review was conducted using the strategy outlined below. The goal was to identify key work that explored how movement sensor technology has been used in healthcare. After an initial selection of key articles, reference lists were screened for additional relevant studies.

Table 1; search strategies

Database	Search terms	Filters	Results
**Medline**	Movement/ or locomotion AND<br>Humans/ or Wearable Electronic Devices/ or Equipment Design/ or Monitoring, Ambulatory/ AND<br>Hospital	Inception – 1.7.2022	16
**ScienceDirect**	"Movement sensor", AND<br>Wearable, AND<br>Health, AND<br>Hospital	Inception – 1.7.2022	128

Survey

To calculate the total number of unique combinations, we multiplied the number of levels (or categories) for each attribute. In our case, this was 4 × 2 × 4 × 3 × 2 × 2 × 4, which equals 1,536 possible combinations (Table 2).

Table 2: Number of levels per attribute

Attribute	Number of levels
Time	4
Complexity	2
Accuracy	4
Sensor type	3
Attachment type	2
Reusability	2
Number of attachments	4

Survey Material

Start of Block: Intro Block

Q1 HIVE of Activity - Survey This study has approval from the Human Research Ethics Committee from the East Metropolitan Health Service (RGS5189) and Curtin University (HREC2023-0118). This study looks at using new technology in hospitals to measure movements in patients who are unwell. There is a relationship between 'movement' and 'health' in people who are admitted to hospital. Better health outcomes are observed in patients who demonstrate an increase in purposeful movement (e.g. walking around) and worse health outcomes tend to be observed in those who demonstrate a reduction in purposeful movements (e.g. bed rest) OR increased non-purposeful movements (e.g. fidgeting). New technology could be used to capture and analyse movement in people admitted to hospital, which may allow clinicians to recognise patterns in movement and assist in making decisions about a patient's care. 'Movement' could be used as a vital sign in the same way that we use heart rate or blood pressure. The purpose of this survey is to develop a consensus opinion - formed by consumers - on what factors influence a consumer's acceptance of wearable sensors in the hospital environment. The survey consists of 10 scenarios and takes approximately 10 minutes to complete. On each page there are two columns with examples of different devices that could be used and ways of attaching them to the patients. Choose the column that describes your preferred option of the two by selecting the button below the column. For survey support, please contact Kylie Hill (k.hill@curtin.edu.au) or Meg Harrold (m.harrold@curtin.edu.au).

Page Break

To participate in this survey, you must be 18 years or over. How old are you (in years)?

- ○ Under 18 (8)
- ○ 18 - 24 years (1)
- ○ 25 - 34 years (2)
- ○ 35 - 44 years (3)
- ○ 45 - 54 years (4)
- ○ 55 - 64 years (5)
- ○ 65+ years (6)

Skip To: End of Block If To participate in this survey, you must be 18 years or over. How old are you (in years)? = Under 18

Page Break

Have you stayed overnight in a hospital in Australia within the last five years (minimum 1-night):

○ Yes (1)

○ No (2)

Skip To: End of Block If Have you stayed overnight in a hospital in Australia within the last five years (minimum 1-night): = No

End of Block: Intro Block

Start of Block: Block 1

*

Group 1

Please read each option and indicate your preferred device by clicking **either** Option 1 or Option 2

	Option 1	Option 2
**Setting up the movement sensor takes this long**	15 minutes	2 minutes
**Setting up the movement sensor is this complex**	• Simple to set up<br>• Set up by a consumer or carer independently	• Difficult to set up<br>• Set up by a health professional only
**When measuring your movement, the sensor system is occasionally inaccurate**	• Inaccurate in 1 out of 100 cases<br>• You are interrupted unnecessarily by a health professional approximately once every 24 hours	• Inaccurate in 1 out of 20 cases<br>• You are interrupted unnecessarily by a health professional approximately once every 6 hours
**The movement sensor system includes the following components**	• A wearable device and no camera<br>• The wearable data can be used to create an avatar that represents the user, but cannot be identified (see image below)<br>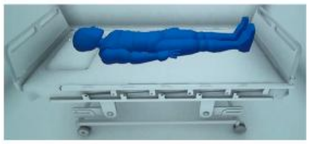	• A camera<br>• The camera is used to capture video footage of the user (see image below)<br>• The footage is kept securely and can only be viewed by a health professional<br>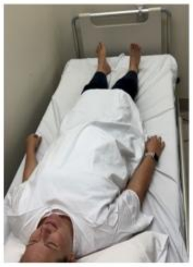
**The wearable movement sensor is attached to your skin in the following way**	• Re-useable, adjustable band<br>• No skin preparation is required to attach the device<br>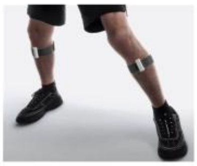	• Temporarily stuck to the skin using an adhesive<br>• The skin must be shaved to ensure the adhesive sticks<br>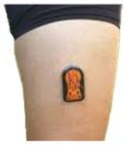
**The wearable movement sensor is discarded in the following conditions**	• Sensor is reusable<br>• When it is removed to shower it can be reapplied afterwards	• Sensor is single use only<br>• When it is removed to shower it must be replaced with a new device afterwards
**The wearable movement sensor requires theses attachments to be worn**	The wearable sensor has 4 attachments worn on the:<br>1. right wrist<br>2. left wrist<br>3. right ankle<br>4. left ankle	The wearable sensor has 2 attachments worn on the:<br>1. middle of the chest<br>2. right **or** left wrist
	Option 1<br>○	Option 2<br>○

Page Break

	Option 1	Option 2
**Setting up the movement sensor takes this long**	2 minutes	15 minutes
**Setting up the movement sensor is this complex**	• Simple to set up<br>• Set up by a consumer or carer independently	• Difficult to set up<br>• Set up by a health professional only
**When measuring your movement, the sensor system is occasionally inaccurate**	• Inaccurate in 1 out of 50 cases<br>• You are interrupted unnecessarily by a health professional approximately once every 12 hours	• Inaccurate in 1 out of 5 cases<br>• You are interrupted unnecessarily by a health professional approximately once every hour
**The movement sensor system includes the following components**	• A wearable device and no camera<br>• The wearable data can be used to create an avatar that represents the user, but cannot be identified (see image below)<br>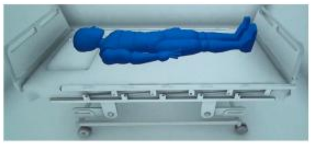	• A thermal camera<br>• Does not capture video footage<br>• Captures unidentifiable images of the user (see image below)<br>• The footage is kept securely and can only be viewed by a health professional<br>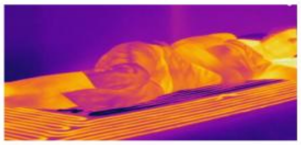
**The wearable movement sensor is attached to your skin in the following way**	• Temporarily stuck to the skin using an adhesive<br>• The skin must be shaved to ensure the adhesive sticks<br>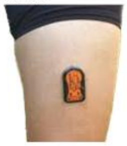	• Re-useable, adjustable band<br>• No skin preparation is required to attach the device<br>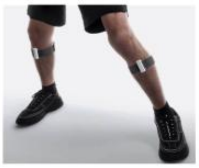
**The wearable movement sensor is discarded in the following conditions**	• Sensor is reusable<br>• When it is removed to shower it can be reapplied afterwards	• Sensor is single use only<br>• When it is removed to shower it must be replaced with a new device afterwards
**The wearable movement sensor requires theses attachments to be worn**	The wearable sensor has 6 attachments worn on the:<br>1. right wrist<br>2. left wrist<br>3. right ankle<br>4. left ankle<br>5. middle of the chest<br>6. forehead	The wearable sensor has 4 attachments worn on the:<br>1. right wrist<br>2. left wrist<br>3. right ankle<br>4. left ankle
	Option 1<br>○	Option 2<br>○

Page Break

	Option 1	Option 2
**Setting up the movement sensor takes this long**	15 minutes	5 minutes
**Setting up the movement sensor is this complex**	• Difficult to set up<br>• Set up by a health professional only	• Simple to set up<br>• Set up by a consumer or carer independently
**When measuring your movement, the sensor system is occasionally inaccurate**	• Inaccurate in 1 out of 5 cases<br>• You are interrupted unnecessarily by a health professional approximately once every hour	• Inaccurate in 1 out of 20 cases<br>• You are interrupted unnecessarily by a health professional approximately once every 6 hours
**The movement sensor system includes the following components**	• A thermal camera<br>• Does not capture video footage<br>• Captures unidentifiable images of the user (see image below)<br>• The footage is kept securely and can only be viewed by a health professional<br>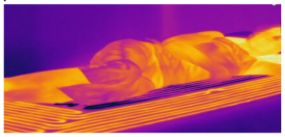	• A wearable device and no camera<br>• The wearable data can be used to create an avatar that represents the user, but cannot be identified (see image below)<br>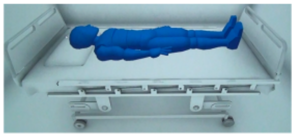
**The wearable movement sensor is attached to your skin in the following way**	• Re-useable, adjustable band<br>• No skin preparation is required to attach the device<br>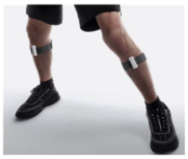	• Temporarily stuck to the skin using an adhesive<br>• The skin must be shaved to ensure the adhesive sticks<br>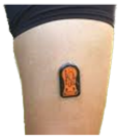
**The wearable movement sensor is discarded in the following conditions**	• Sensor is single use only<br>• When it is removed to shower it must be replaced with a new device afterwards	• Sensor is reusable<br>• When it is removed to shower it can be reapplied afterwards
**The wearable movement sensor requires theses attachments to be worn**	The wearable sensor has 2 attachments worn on the:<br>1. middle of the chest<br>2. forehead	The wearable sensor has 4 attachments worn on the:<br>1. right wrist<br>2. left wrist<br>3. right ankle<br>4. left ankle

Option 1	Option 2
○	○

Page Break

	Option 1	Option 2
**Setting up the movement sensor takes this long**	5 minutes	10 minutes
**Setting up the movement sensor is this complex**	• Simple to set up<br>• Set up by a consumer or carer independently	• Difficult to set up<br>• Set up by a health professional only
**When measuring your movement, the sensor system is occasionally inaccurate**	• Inaccurate in 1 out of 50 cases<br>• You are interrupted unnecessarily by a health professional approximately once every 12 hours	• Inaccurate in 1 out of 100 cases<br>• You are interrupted unnecessarily by a health professional approximately once every 24 hours
**The movement sensor system includes the following components**	• A camera<br>• The camera is used to capture video footage of the user (see image below)<br>• The footage is kept securely and can only be viewed by a health professional<br>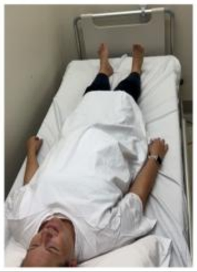	• A wearable device and no camera<br>• The wearable data can be used to create an avatar that represents the user, but cannot be identified (see image below)<br>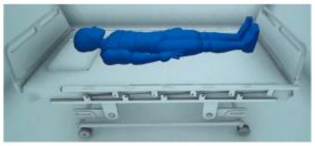
**The wearable movement sensor is attached to your skin in the following way**	• Re-useable, adjustable band<br>• No skin preparation is required to attach the device<br>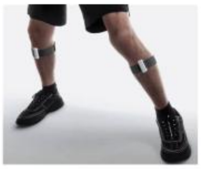	• Temporarily stuck to the skin using an adhesive<br>• The skin must be shaved to ensure the adhesive sticks<br>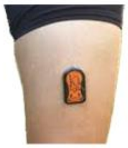
**The wearable movement sensor is discarded in the following conditions**	• Sensor is single use only<br>• When it is removed to shower it must be replaced with a new device afterwards	• Sensor is reusable<br>• When it is removed to shower it can be reapplied afterwards
**The wearable movement sensor requires these attachments to be worn**	The wearable sensor has 6 attachments worn on the:<br>1. right wrist<br>2. left wrist<br>3. right ankle<br>4. left ankle<br>5. middle of the chest<br>6. forehead	The wearable sensor has 2 attachments worn on the:<br>1. middle of the chest<br>2. right **or** left wrist
	Option 1<br>○	Option 2<br>○

	Option 1	Option 2
**Setting up the movement sensor takes this long**	15 minutes	5 minutes
**Setting up the movement sensor is this complex**	• Simple to set up<br>• Set up by a consumer or carer independently	• Difficult to set up<br>• Set up by a health professional only
**When measuring your movement, the sensor system is occasionally inaccurate**	• Inaccurate in 1 out of 100 cases<br>• You are interrupted unnecessarily by a health professional approximately once every 24 hours	• Inaccurate in 1 out of 20 cases<br>• You are interrupted unnecessarily by a health professional approximately once every 6 hours
**The movement sensor system includes the following components**	• A camera<br>• The camera is used to capture video footage of the user (see image below)<br>• The footage is kept securely and can only be viewed by a health professional<br>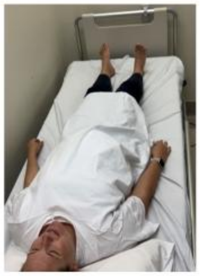	• A thermal camera<br>• Does not capture video footage<br>• Captures unidentifiable images of the user (see image below)<br>• The footage is kept securely and can only be viewed by a health professional<br>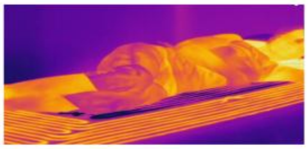
**The wearable movement sensor is attached to your skin in the following way**	• Temporarily stuck to the skin using an adhesive<br>• The skin must be shaved to ensure the adhesive sticks<br>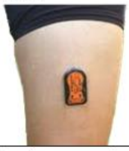	• Re-useable, adjustable band<br>• No skin preparation is required to attach the device<br>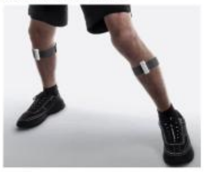
**The wearable movement sensor is discarded in the following conditions**	• Sensor is single use only<br>• When it is removed to shower it must be replaced with a new device afterwards	• Sensor is reusable<br>• When it is removed to shower it can be reapplied afterwards
**The wearable movement sensor requires these attachments to be worn**	The wearable sensor has 2 attachments worn on the:<br>1. middle of the chest<br>2. right **or** left wrist	The wearable sensor has 2 attachments worn on the:<br>1. middle of the chest<br>2. forehead

	Option 1	Option 2
	○	○

	Option 1	Option 2
**Setting up the movement sensor takes this long**	5 minutes	2 minutes
**Setting up the movement sensor is this complex**	• Simple to set up<br>• Set up by a consumer or carer independently	• Difficult to set up<br>• Set up by a health professional only
**When measuring your movement, the sensor system is occasionally inaccurate**	• Inaccurate in 1 out of 100 cases<br>• You are interrupted unnecessarily by a health professional approximately once every 24 hours	• Inaccurate in 1 out of 5 cases<br>• You are interrupted unnecessarily by a health professional approximately once every hour
**The movement sensor system includes the following components**	• A thermal camera<br>• Does not capture video footage<br>• Captures unidentifiable images of the user (see image below)<br>• The footage is kept securely and can only be viewed by a health professional<br>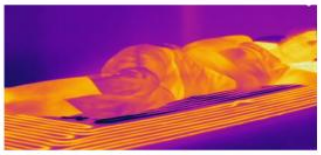	• A wearable device and no camera<br>• The wearable data can be used to create an avatar that represents the user, but cannot be identified (see image below)<br>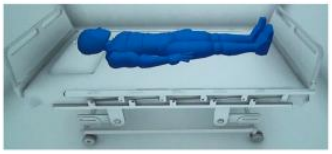
**The wearable movement sensor is attached to your skin in the following way**	• Temporarily stuck to the skin using an adhesive<br>• The skin must be shaved to ensure the adhesive sticks<br>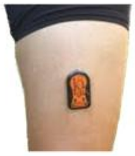	• Re-useable, adjustable band<br>• No skin preparation is required to attach the device<br>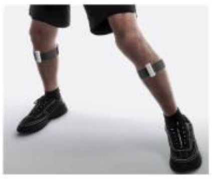
**The wearable movement sensor is discarded in the following conditions**	• Sensor is single use only<br>• When it is removed to shower it must be replaced with a new device afterwards	• Sensor is reusable<br>• When it is removed to shower it can be reapplied afterwards
**The wearable movement sensor requires these attachments to be worn**	The wearable sensor has 2 attachments worn on the:<br>1. middle of the chest<br>2. right **or** left wrist	The wearable sensor has 6 attachments worn on the:<br>1. right wrist<br>2. left wrist<br>3. right ankle<br>4. left ankle<br>5. middle of the chest<br>6. forehead
	Option 1<br>○	Option 2<br>○

Page Break

	Option 1	Option 2
**Setting up the movement sensor takes this long**	5 minutes	2 minutes
**Setting up the movement sensor is this complex**	• Difficult to set up<br>• Set up by a health professional only	• Simple to set up<br>• Set up by a consumer or carer independently
**When measuring your movement, the sensor system is occasionally inaccurate**	• Inaccurate in 1 out of 5 cases<br>• You are interrupted unnecessarily by a health professional approximately once every hour	• Inaccurate in 1 out of 50 cases<br>• You are interrupted unnecessarily by a health professional approximately once every 12 hours
**The movement sensor system includes the following components**	• A wearable device and no camera<br>• The wearable data can be used to create an avatar that represents the user, but cannot be identified (see image below)<br>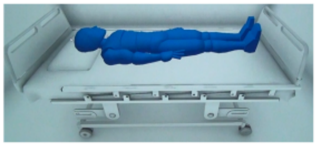	• A camera<br>• The camera is used to capture video footage of the user (see image below)<br>• The footage is kept securely and can only be viewed by a health professional<br>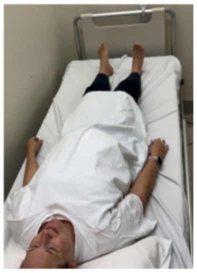
**The wearable movement sensor is attached to your skin in the following way**	• Temporarily stuck to the skin using an adhesive<br>• The skin must be shaved to ensure the adhesive sticks<br>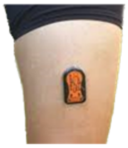	• Re-useable, adjustable band<br>• No skin preparation is required to attach the device<br>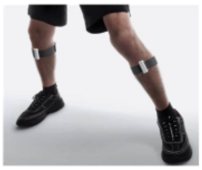
**The wearable movement sensor is discarded in the following conditions**	• Sensor is single use only<br>• When it is removed to shower it must be replaced with a new device afterwards	• Sensor is reusable<br>• When it is removed to shower it can be reapplied afterwards
**The wearable movement sensor requires these attachments to be worn**	The wearable sensor has 6 attachments worn on the:<br>1. right wrist<br>2. left wrist<br>3. right ankle<br>4. left ankle<br>5. middle of the chest<br>6. forehead	The wearable sensor has 1 attachment worn on the:<br>1. middle of the chest

Option 1 ○ Option 2 ○

	Option 1	Option 2
**Setting up the movement sensor takes this long**	2 minutes	10 minutes
**Setting up the movement sensor is this complex**	• Difficult to set up<br>• Set up by a health professional only	• Simple to set up<br>• Set up by a consumer or carer independently
**When measuring your movement, the sensor system is occasionally inaccurate**	• Inaccurate in 1 out of 100 cases<br>• You are interrupted unnecessarily by a health professional approximately once every 24 hours	• Inaccurate in 1 out of 5 cases<br>• You are interrupted unnecessarily by a health professional approximately once every hour
**The movement sensor system includes the following components**	• A wearable device and no camera<br>• The wearable data can be used to create an avatar that represents the user, but cannot be identified (see image below)<br>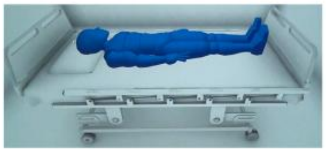	• A camera<br>• The camera is used to capture video footage of the user (see image below)<br>• The footage is kept securely and can only be viewed by a health professional<br>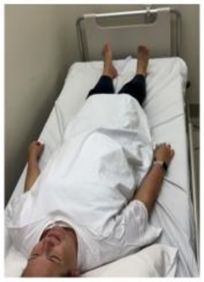
**The wearable movement sensor is attached to your skin in the following way**	• Re-useable, adjustable band<br>• No skin preparation is required to attach the device<br>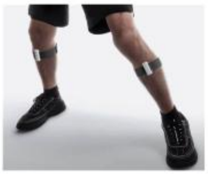	• Temporarily stuck to the skin using an adhesive<br>• The skin must be shaved to ensure the adhesive sticks<br>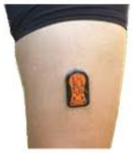
**The wearable movement sensor is discarded in the following conditions**	• Sensor is single use only<br>• When it is removed to shower it must be replaced with a new device afterwards	• Sensor is reusable<br>• When it is removed to shower it can be reapplied afterwards
**The wearable movement sensor requires these attachments to be worn**	The wearable sensor has 2 attachments worn on the:<br>1. middle of the chest<br>2. forehead	The wearable sensor has 6 attachments worn on the:<br>1. right wrist<br>2. left wrist<br>3. right ankle<br>4. left ankle<br>5. middle of the chest<br>6. forehead
	Option 1<br>○	Option 2<br>○

Page Break

	Option 1	Option 2
**Setting up the movement sensor takes this long**	2 minutes	10 minutes
**Setting up the movement sensor is this complex**	• Difficult to set up<br>• Set up by a health professional only	• Simple to set up<br>• Set up by a consumer or carer independently
**When measuring your movement, the sensor system is occasionally inaccurate**	• Inaccurate in 1 out of 20 cases<br>• You are interrupted unnecessarily by a health professional approximately once every 6 hours	• Inaccurate in 1 out of 100 cases<br>• You are interrupted unnecessarily by a health professional approximately once every 24 hours
**The movement sensor system includes the following components**	• A camera<br>• The camera is used to capture video footage of the user (see image below)<br>• The footage is kept securely and can only be viewed by a health professional<br>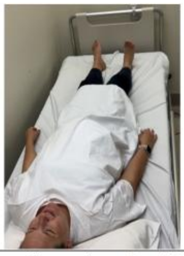	• A thermal camera<br>• Does not capture video footage<br>• Captures unidentifiable images of the user (see image below)<br>• The footage is kept securely and can only be viewed by a health professional<br>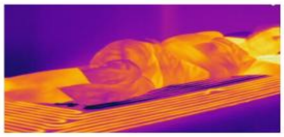
**The wearable movement sensor is attached to your skin in the following way**	• Temporarily stuck to the skin using an adhesive<br>• The skin must be shaved to ensure the adhesive sticks<br>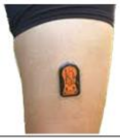	• Re-useable, adjustable band<br>• No skin preparation is required to attach the device<br>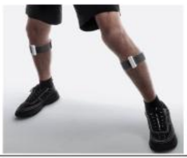
**The wearable movement sensor is discarded in the following conditions**	• Sensor is single use only<br>• When it is removed to shower it must be replaced with a new device afterwards	• Sensor is reusable<br>• When it is removed to shower it can be reapplied afterwards
**The wearable movement sensor requires these attachments to be worn**	The wearable sensor has 4 attachments worn on the:<br>1. right wrist<br>2. left wrist<br>3. right ankle<br>4. left ankle	The wearable sensor has 1 attachment worn on the:<br>1. middle of the chest

	Option 1	Option 2
	○	○

Page Break

	Option 1	Option 2
**Setting up the movement sensor takes this long**	15 minutes	10 minutes
**Setting up the movement sensor is this complex**	• Simple to set up<br>• Set up by a consumer or carer independently	• Difficult to set up<br>• Set up by a health professional only
**When measuring your movement, the sensor system is occasionally inaccurate**	• Inaccurate in 1 out of 20 cases<br>• You are interrupted unnecessarily by a health professional approximately once every 6 hours	• Inaccurate in 1 out of 100 cases<br>• You are interrupted unnecessarily by a health professional approximately once every 24 hours
**The movement sensor system includes the following components**	• A thermal camera<br>• Does not capture video footage<br>• Captures unidentifiable images of the user (see image below)<br>• The footage is kept securely and can only be viewed by a health professional<br>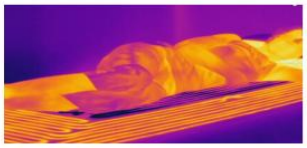	• A camera<br>• The camera is used to capture video footage of the user (see image below)<br>• The footage is kept securely and can only be viewed by a health professional<br>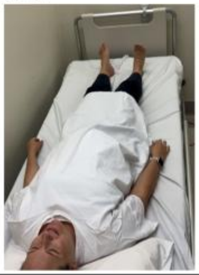
**The wearable movement sensor is attached to your skin in the following way**	• Re-useable, adjustable band<br>• No skin preparation is required to attach the device<br>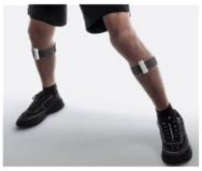	• Temporarily stuck to the skin using an adhesive<br>• The skin must be shaved to ensure the adhesive sticks<br>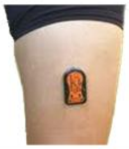
**The wearable movement sensor is discarded in the following conditions**	• Sensor is reusable<br>• When it is removed to shower it can be reapplied afterwards	• Sensor is single use only<br>• When it is removed to shower it must be replaced with a new device afterwards
**The wearable movement sensor requires these attachments to be worn**	The wearable sensor has 2 attachments worn on the:<br>1. middle of the chest<br>2. right **or** left wrist	The wearable sensor has 2 attachments worn on the:<br>1. middle of the chest<br>2. forehead
	Option 1<br>○	Option 2<br>○

End of Block: Block 1

Start of Block: Block 2

*

Group 2

Please read each option and indicate your preferred device by clicking **either** Option 1 or Option 2

	Option 1	Option 2
**Setting up the movement sensor takes this long**	10 minutes	2 minutes
**Setting up the movement sensor is this complex**	• Difficult to set up<br>• Set up by a health professional only	• Simple to set up<br>• Set up by a consumer or carer independently
**When measuring your movement, the sensor system is occasionally inaccurate**	• Inaccurate in 1 out of 50 cases<br>• You are interrupted unnecessarily by a health professional approximately once every 12 hours	• Inaccurate in 1 out of 100 cases<br>• You are interrupted unnecessarily by a health professional approximately once every 24 hours
**The movement sensor system includes the following components**	• A thermal camera<br>• Does not capture video footage<br>• Captures unidentifiable images of the user (see image below)<br>• The footage is kept securely and can only be viewed by a health professional<br>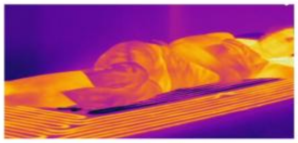	• A camera<br>• The camera is used to capture video footage of the user (see image below)<br>• The footage is kept securely and can only be viewed by a health professional<br>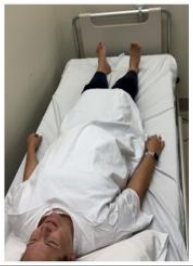
**The wearable movement sensor is attached to your skin in the following way**	• Temporarily stuck to the skin using an adhesive<br>• The skin must be shaved to ensure the adhesive sticks<br>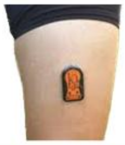	• Re-useable, adjustable band<br>• No skin preparation is required to attach the device<br>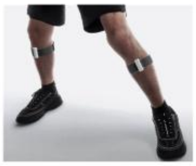
**The wearable movement sensor is discarded in the following conditions**	• Sensor is reusable<br>• When it is removed to shower it can be reapplied afterwards	• Sensor is single use only<br>• When it is removed to shower it must be replaced with a new device afterwards
**The wearable movement sensor requires these attachments to be worn**	The wearable sensor has 2 attachments worn on the:<br>1· middle of the chest<br>2· forehead	The wearable sensor has 4 attachments worn on the:<br>1· right wrist<br>2· left wrist<br>3· right ankle<br>4· left ankle
	Option 1<br>○	Option 2<br>○

Page Break

	Option 1	Option 2
**Setting up the movement sensor takes this long**	10 minutes	5 minutes
**Setting up the movement sensor is this complex**	• Difficult to set up<br>• Set up by a health professional only	• Simple to set up<br>• Set up by a consumer or carer independently
**When measuring your movement, the sensor system is occasionally inaccurat**	• Inaccurate in 1 out of 50 cases<br>• You are interrupted unnecessarily by a health professional approximately once every 12 hours	• Inaccurate in 1 out of 5 cases<br>• You are interrupted unnecessarily by a health professional approximately once every hour
**The movement sensor system includes the following components**	• A wearable device and no camera<br>• The wearable data can be used to create an avatar that represents the user, but cannot be identified (see image below)<br>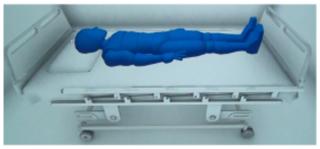	• A thermal camera<br>• Does not capture video footage<br>• Captures unidentifiable images of the user (see image below)<br>• The footage is kept securely and can only be viewed by a health professional<br>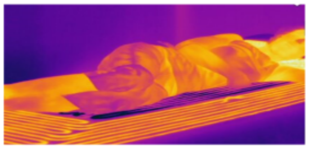
**The wearable movement sensor is attached to your skin in the following way**	• Re-useable, adjustable band<br>• No skin preparation is required to attach the device<br>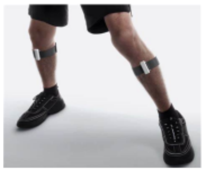	• Temporarily stuck to the skin using an adhesive<br>• The skin must be shaved to ensure the adhesive sticks<br>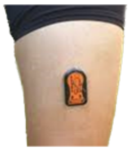
**The wearable movement sensor is discarded in the following conditions**	• Sensor is single use only<br>• When it is removed to shower it must be replaced with a new device afterwards	• Sensor is reusable<br>• When it is removed to shower it can be reapplied afterwards
**The wearable movement sensor requires these attachments to be worn**	The wearable sensor has 2 attachments worn on the:<br>1. middle of the chest<br>2. right **or** left wrist	The wearable sensor has 2 attachments worn on the:<br>1. middle of the chest<br>2. forehead

Option 1 ○ Option 2 ○

Page Break

	Option 1	Option 2
**Setting up the movement sensor takes this long**	2 minutes	5 minutes
**Setting up the movement sensor is this complex**	• Simple to set up<br>• Set up by a consumer or carer independently	• Difficult to set up<br>• Set up by a health professional only
**When measuring your movement, the sensor system is occasionally inaccurate**	• Inaccurate in 1 out of 5 cases<br>• You are interrupted unnecessarily by a health professional approximately once every hour	• Inaccurate in 1 out of 20 cases<br>• You are interrupted unnecessarily by a health professional approximately once every 6 hours
**The movement sensor system includes the following components**	• A thermal camera<br>• Does not capture video footage<br>• Captures unidentifiable images of the user (see image below)<br>• The footage is kept securely and can only be viewed by a health professional<br>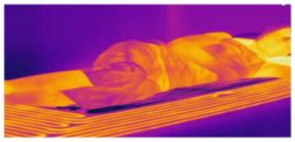	• A camera<br>• The camera is used to capture video footage of the user (see image below)<br>• The footage is kept securely and can only be viewed by a health professional<br>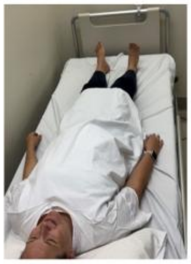
**The wearable movement sensor is attached to your skin in the following way**	• Temporarily stuck to the skin using an adhesive<br>• The skin must be shaved to ensure the adhesive sticks<br>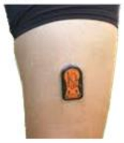	• Re-useable, adjustable band<br>• No skin preparation is required to attach the device<br>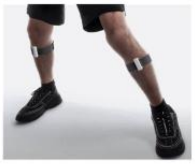
**The wearable movement sensor is discarded in the following conditions**	• Sensor is reusable<br>• When it is removed to shower it can be reapplied afterwards	• Sensor is single use only<br>• When it is removed to shower it must be replaced with a new device afterwards
**The wearable movement sensor requires these attachments to be worn**	The wearable sensor has 4 attachments worn on the:<br>1. right wrist<br>2. left wrist<br>3. right ankle<br>4. left ankle	The wearable sensor has 6 attachments worn on the:<br>1. right wrist<br>2. left wrist<br>3. right ankle<br>4. left ankle<br>5. middle of the chest<br>6. forehead
	Option 1<br>○	Option 2<br>○

Page Break

	Option 1	Option 2
**Setting up the movement sensor takes this long**	15 minutes	2 minutes
**Setting up the movement sensor is this complex**	• Difficult to set up<br>• Set up by a health professional only	• Simple to set up<br>• Set up by a consumer or carer independently
**When measuring your movement, the sensor system is occasionally inaccurate**	• Inaccurate in 1 out of 20 cases<br>• You are interrupted unnecessarily by a health professional approximately once every 6 hours	• Inaccurate in 1 out of 5 cases<br>• You are interrupted unnecessarily by a health professional approximately once every hour
**The movement sensor system includes the following components**	• A camera<br>• The camera is used to capture video footage of the user (see image below)<br>• The footage is kept securely and can only be viewed by a health professional<br>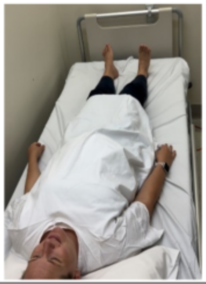	• A wearable device and no camera<br>• The wearable data can be used to create an avatar that represents the user, but cannot be identified (see image below)<br>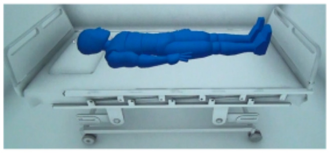
**The wearable movement sensor is attached to your skin in the following way**	• Temporarily stuck to the skin using an adhesive<br>• The skin must be shaved to ensure the adhesive sticks<br>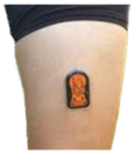	• Re-useable, adjustable band<br>• No skin preparation is required to attach the device<br>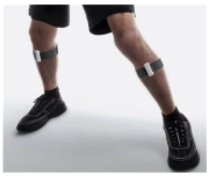
**The wearable movement sensor is discarded in the following conditions**	• Sensor is reusable<br>• When it is removed to shower it can be reapplied afterwards	• Sensor is single use only<br>• When it is removed to shower it must be replaced with a new device afterwards
**The wearable movement sensor requires these attachments to be worn**	The wearable sensor has 1 attachment worn on the:<br>1· middle of the chest	The wearable sensor has 2 attachments worn on the:<br>1· middle of the chest<br>2· right **or** left wrist

Option 1 ○ Option 2 ○

	Option 1	Option 2
**Setting up the movement sensor takes this long**	2 minutes	15 minutes
**Setting up the movement sensor is this complex**	• Difficult to set up<br>• Set up by a health professional only	• Simple to set up<br>• Set up by a consumer or carer independently
**When measuring your movement, the sensor system is occasionally inaccurate**	• Inaccurate in 1 out of 100 cases<br>• You are interrupted unnecessarily by a health professional approximately once every 24 hours	• Inaccurate in 1 out of 50 cases<br>• You are interrupted unnecessarily by a health professional approximately once every 12 hours
**The movement sensor system includes the following components**	• A thermal camera<br>• Does not capture video footage<br>• Captures unidentifiable images of the user (see image below)<br>• The footage is kept securely and can only be viewed by a health professional<br>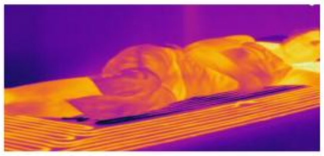	• A wearable device and no camera<br>• The wearable data can be used to create an avatar that represents the user, but cannot be identified (see image below)<br>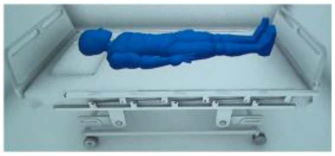
**The wearable movement sensor is attached to your skin in the following way**	• Temporarily stuck to the skin using an adhesive<br>• The skin must be shaved to ensure the adhesive sticks<br>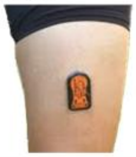	• Re-useable, adjustable band<br>• No skin preparation is required to attach the device<br>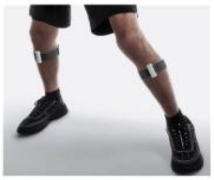
**The wearable movement sensor is discarded in the following conditions**	• Sensor is reusable<br>• When it is removed to shower it can be reapplied afterwards	• Sensor is single use only<br>• When it is removed to shower it must be replaced with a new device afterwards
**The wearable movement sensor requires these attachments to be worn**	The wearable sensor has 6 attachments worn on the:<br>1· right wrist<br>2· left wrist<br>3· right ankle<br>4· left ankle<br>5· middle of the chest<br>6· forehead	The wearable sensor has 2 attachments worn on the:<br>1· middle of the chest<br>2· forehead
	Option 1<br>○	Option 2<br>○

	Option 1	Option 2
**Setting up the movement sensor takes this long**	10 minutes	15 minutes
**Setting up the movement sensor is this complex**	• Simple to set up<br>• Set up by a consumer or carer independently	• Difficult to set up<br>• Set up by a health professional only
**When measuring your movement, the sensor system is occasionally inaccurate**	• Inaccurate in 1 out of 20 cases<br>• You are interrupted unnecessarily by a health professional approximately once every 6 hours	• Inaccurate in 1 out of 50 cases<br>• You are interrupted unnecessarily by a health professional approximately once every 12 hours
**The movement sensor system includes the following components**	• A thermal camera<br>• Does not capture video footage<br>• Captures unidentifiable images of the user (see image below)<br>• The footage is kept securely and can only be viewed by a health professional<br>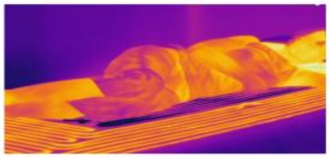	• A camera<br>• The camera is used to capture video footage of the user (see image below)<br>• The footage is kept securely and can only be viewed by a health professional<br>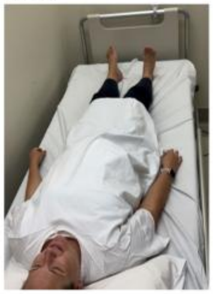
**The wearable movement sensor is attached to your skin in the following way**	• Re-useable, adjustable band<br>• No skin preparation is required to attach the device<br>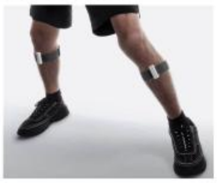	• Temporarily stuck to the skin using an adhesive<br>• The skin must be shaved to ensure the adhesive sticks<br>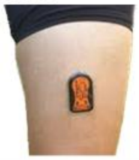
**The wearable movement sensor is discarded in the following conditions**	• Sensor is single use only<br>• When it is removed to shower it must be replaced with a new device afterwards	• Sensor is reusable<br>• When it is removed to shower it can be reapplied afterwards
**The wearable movement sensor requires these attachments to be worn**	The wearable sensor has 1 attachment worn on the:<br>1. middle of the chest	The wearable sensor has 2 attachments worn on the:<br>1. middle of the chest<br>2. right **or** left wrist
	Option 1<br>○	Option 2<br>○

	Option 1	Option 2
**Setting up the movement sensor takes this long**	10 minutes	15 minutes
**Setting up the movement sensor is this complex**	• Simple to set up<br>• Set up by a consumer or carer independently	• Difficult to set up<br>• Set up by a health professional only
**When measuring your movement, the sensor system is occasionally inaccurate**	• Inaccurate in 1 out of 5 cases<br>• You are interrupted unnecessarily by a health professional approximately once every hour	• Inaccurate in 1 out of 100 cases<br>• You are interrupted unnecessarily by a health professional approximately once every 24 hours
**The movement sensor system includes the following components**	• A wearable device and no camera<br>• The wearable data can be used to create an avatar that represents the user, but cannot be identified (see image below)<br>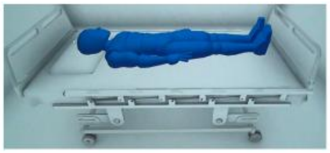	• A thermal camera<br>• Does not capture video footage<br>• Captures unidentifiable images of the user (see image below)<br>• The footage is kept securely and can only be viewed by a health professional<br>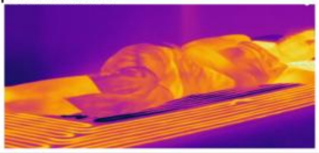
**The wearable movement sensor is attached to your skin in the following way**	• Temporarily stuck to the skin using an adhesive<br>• The skin must be shaved to ensure the adhesive sticks<br>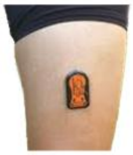	• Re-useable, adjustable band<br>• No skin preparation is required to attach the device<br>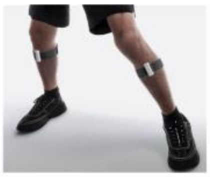
**The wearable movement sensor is discarded in the following conditions**	• Sensor is single use only<br>• When it is removed to shower it must be replaced with a new device afterwards	• Sensor is reusable<br>• When it is removed to shower it can be reapplied afterwards
**The wearable movement sensor requires these attachments to be worn**	The wearable sensor has 1 attachment worn on the:<br>1· middle of the chest	The wearable sensor has 6 attachments worn on the:<br>1· right wrist<br>2· left wrist<br>3· right ankle<br>4· left ankle<br>5· middle of the chest<br>6· forehead
	Option 1<br>○	Option 2<br>○

	Option 1	Option 2
**Setting up the movement sensor takes this long**	5 minutes	15 minutes
**Setting up the movement sensor is this complex**	• Difficult to set up<br>• Set up by a health professional only	• Simple to set up<br>• Set up by a consumer or carer independently
**When measuring your movement, the sensor system is occasionally inaccurate**	• Inaccurate in 1 out of 50 cases<br>• You are interrupted unnecessarily by a health professional approximately once every 12 hours	• Inaccurate in 1 out of 20 cases<br>• You are interrupted unnecessarily by a health professional approximately once every 6 hours
**The movement sensor system includes the following components**	• A camera<br>• The camera is used to capture video footage of the user (see image below)<br>• The footage is kept securely and can only be viewed by a health professional<br>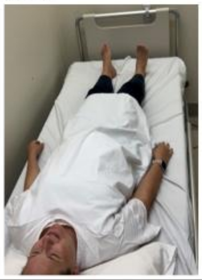	• A wearable device and no camera<br>• The wearable data can be used to create an avatar that represents the user, but cannot be identified (see image below)<br>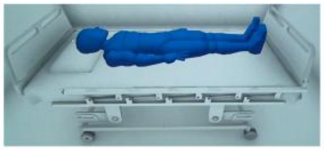
**The wearable movement sensor is attached to your skin in the following way**	• Re-useable, adjustable band<br>• No skin preparation is required to attach the device<br>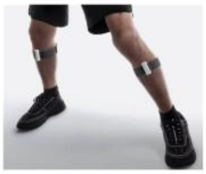	• Temporarily stuck to the skin using an adhesive<br>• The skin must be shaved to ensure the adhesive sticks<br>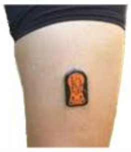
**The wearable movement sensor is discarded in the following conditions**	• Sensor is reusable<br>• When it is removed to shower it can be reapplied afterwards	• Sensor is single use only<br>• When it is removed to shower it must be replaced with a new device afterwards
**The wearable movement sensor requires these attachments to be worn**	The wearable sensor has 4 attachments worn on the:<br>1. right wrist<br>2. left wrist<br>3. right ankle<br>4. left ankle	The wearable sensor has 1 attachment worn on the:<br>1. middle of the chest
	Option 1<br>○	Option 2<br>○

	Option 1	Option 2
**Setting up the movement sensor takes this long**	5 minutes	10 minutes
**Setting up the movement sensor is this complex**	• Difficult to set up<br>• Set up by a health professional only	• Simple to set up<br>• Set up by a consumer or carer independently
**When measuring your movement, the sensor system is occasionally inaccurate**	• Inaccurate in 1 out of 5 cases<br>• You are interrupted unnecessarily by a health professional approximately once every hour	• Inaccurate in 1 out of 50 cases<br>• You are interrupted unnecessarily by a health professional approximately once every 12 hours
**The movement sensor system includes the following components**	• A camera<br>• The camera is used to capture video footage of the user (see image below)<br>• The footage is kept securely and can only be viewed by a health professional<br>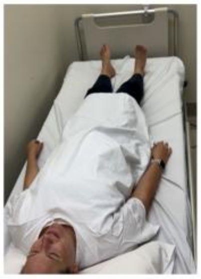	• A thermal camera<br>• Does not capture video footage<br>• Captures unidentifiable images of the user (see image below)<br>• The footage is kept securely and can only be viewed by a health professional<br>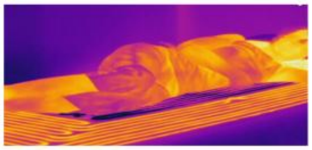
**The wearable movement sensor is attached to your skin in the following way**	• Re-useable, adjustable band<br>• No skin preparation is required to attach the device<br>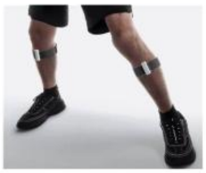	• Temporarily stuck to the skin using an adhesive<br>• The skin must be shaved to ensure the adhesive sticks<br>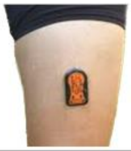
**The wearable movement sensor is discarded in the following conditions**	• Sensor is reusable<br>• When it is removed to shower it can be reapplied afterwards	• Sensor is single use only<br>• When it is removed to shower it must be replaced with a new device afterwards
**The wearable movement sensor requires these attachments to be worn**	The wearable sensor has 1 attachment worn on the:<br>1. middle of the chest	The wearable sensor has 4 attachments worn on the:<br>1. right wrist<br>2. left wrist<br>3. right ankle<br>4. left ankle
	Option 1<br>○	Option 2<br>○

	Option 1	Option 2
**Setting up the movement sensor takes this long**	10 minutes	5 minutes
**Setting up the movement sensor is this complex**	• Simple to set up<br>• Set up by a consumer or carer independently	• Difficult to set up<br>• Set up by a health professional only
**When measuring your movement, the sensor system is occasionally inaccurate**	• Inaccurate in 1 out of 20 cases<br>• You are interrupted unnecessarily by a health professional approximately once every 6 hours	• Inaccurate in 1 out of 50 cases<br>• You are interrupted unnecessarily by a health professional approximately once every 12 hours
**The movement sensor system includes the following components**	• A camera<br>• The camera is used to capture video footage of the user (see image below)<br>• The footage is kept securely and can only be viewed by a health professional<br>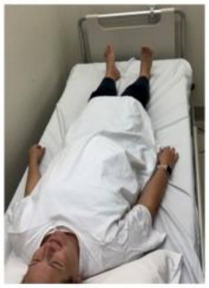	• A thermal camera<br>• Does not capture video footage<br>• Captures unidentifiable images of the user (see image below)<br>• The footage is kept securely and can only be viewed by a health professional<br>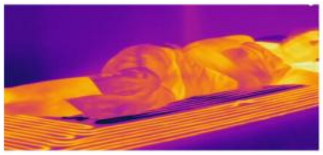
**The wearable movement sensor is attached to your skin in the following way**	• Re-useable, adjustable band<br>• No skin preparation is required to attach the device<br>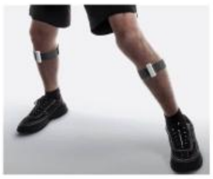	• Temporarily stuck to the skin using an adhesive<br>• The skin must be shaved to ensure the adhesive sticks<br>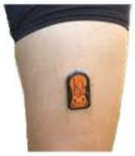
**The wearable movement sensor is discarded in the following conditions**	• Sensor is reusable<br>• When it is removed to shower it can be reapplied afterwards	• Sensor is single use only<br>• When it is removed to shower it must be replaced with a new device afterwards
**The wearable movement sensor requires these attachments to be worn**	The wearable sensor has 2 attachments worn on the:<br>1. middle of the chest<br>2. forehead	The wearable sensor has 1 attachment worn on the:<br>1. middle of the chest

	Option 1	Option 2
	○	○

End of Block: Block 2

Clinical Focus Group Prompts

'A HIVE OF ACTIVITY': FACTORS THAT INFLUENCE THE MEASUREMENT OF MOVEMENT IN HOSPITAL PATIENTS – A QUALITATIVE EXPLORATION CAPTURING THE VOICE OF CLINICIANS AND CONSUMERS.

Topic	Content	Timing
**Introduction**	• Confirm consent (start recording)<br>• Research aims / why<br>• Showcase technology	5 minutes
**Discussion 1**<br><br>**Important movements**	• How do you use movement in your clinical assessment?<br>• How do you assess movement in your clinical assessment?<br>• What factors do use to describe movement to your colleges?	20 min
**Discussion 2**<br>**Technology Acceptance**	• What are the barriers to using monitors in hospital today?<br>• Tell me about some examples of technology that works well?	20 min
**Discussion 3**<br><br>**RedCAP demo**	• Here is a list of movements and positions that the literature suggests are important:<br>o Assessing the patient's ability to move their upper limbs through a full and normal pattern of movement<br>o Assessing the patient's ability to move their lower limbs through a full and normal pattern of movement<br>o Assessing the position of the trunk in bed (for example, the patient is slumped, the patient is on their side)<br>o Assessing the symmetry of movement between the upper limbs<br>o Assessing the symmetry of movement between the low limbs<br>o Assessing the orientation of the patient relative to the bed (for example, the patient is lying diagonally in the bed, the patient is sitting up in bed)<br>o Assessing the speed at which the patient can move or change positions<br>Do you feel this list is complete and or would you like to add any additional items?<br>• How easy is this form to use?	10 minutes
**Conclusion**	• Summary of discussion	5 minutes

Consumer Focus Group – Design Prompt

Discrete Choice Experiment - survey design

		Option 0	Option 1	Option 2	Option 3	Option 4	Comments from Group
**Time**	**Setting up the movement sensor takes this amount of time.**	15 minutes and must be set up by a health professional	10 minutes and must be set up by a health professional	5 minutes and must be set up by a health professional	2 minutes and must be set up by a health professional	2 minutes by a consumer (i.e. doesn't need a health professional to apply)	- Time for consumer with disability<br>- Impact of medical attachments
**Attachment**	**The movement sensor comes with the following attachments**	6 attachments worn as follows:<br>- 1 x right wrist<br>- 1 x left wrist<br>- 1 x right ankle<br>- 1 x left ankle<br>- 1 x trunk<br>- 1 x head	4 attachments worn as follows:<br>- 1 x right wrist<br>- 1 x left wrist<br>- 1 x right ankle<br>- 1 x left ankle	2 attachments<br>- 1 x trunk<br>- 1 x head	2 attachments worn as follows:<br>- 1 x trunk<br>- 1 x wrist	1 attachment worn as follows:<br>- 1 x trunk	
**Wearable**	**The movement sensor is attached to your skin in the following way**	Temporarily stuck to the skin using an adhesive – the skin must be shaved to ensure the adhesive sticks.	Worn using an adjustable band.	N/A	N/A	N/A	
**Remote**	**The movement sensor includes the following additions**	In addition to the movement sensor, a radar camera is used to measure movement. This is NOT attached to the consumer and does not capture an identifiable video image.	There is no additional components - only the movement sensor is used to capture movement.	N/A	N/A	N/A	- Camera description
**Accuracy**	**When measuring your movement, the sensor misclassifies the data**	In 20 out of 100 cases. This results in a relatively high number of unnecessary alarms at the bedside.	In 10 out of 100 cases. This results in a moderate number of unnecessary alarms at the bedside.	In 5 out of 100 cases. This results in a low number of unnecessary alarms at the bedside.	In 1 out of 100 cases. This results in very little unnecessary alarms at the bedside.	N/A	
**Enviro**	The movement sensor is	Not reusable and must be discarded after a single use. Because of this, there is a relatively high financial burden to the hospital and environmental impact.	Reusable and can be reapplied (to the same consumer) after each wear. Because of this, the device has a relatively low financial burden to the hospital and environmental impact.	N/A	N/A	N/A	- Data storage costs
						**Other:**	- Change management resources<br>- Medical staff perception
